# Prevalence and predictors of virological failure in pediatric patients on HAART in sub-Saharan Africa: a systematic review and meta-analysis

**DOI:** 10.11604/pamj.2023.45.98.37017

**Published:** 2023-06-22

**Authors:** Nchimunya Machila, Liyali Libonda, Paul Habineza, Rachel Milomba Velu, Harvey Kakoma Kamboyi, Jacob Ndhlovu, Inonge Wamunyima, Monahani Makwibba Sinadambwe, Steward Mudenda, Cosmas Zyambo, Flavien Nsoni Bumbangi

**Affiliations:** 1 Department of Disease Control and Prevention, School of Medicine, Eden University, Lusaka, Zambia,; 2 Centre for Infectious Disease Research in Zambia, Lusaka, Zambia,; 3 Division of Infection and Immunity, International Institute for Zoonosis Control, Hokkaido University, Hokkaido, Japan,; 4 Department of Women and Newborn, University Teaching Hospital, Lusaka, Zambia,; 5 Department of Pharmacy, School of Medicine, University of Zambia, Lusaka, Zambia,; 6 Department of Community and Family Health, School of Public Health, University of Zambia, Lusaka, Zambia

**Keywords:** Virological failure, HAART, children, pediatrics, prevalence, sub-Saharan Africa

## Abstract

Antiretroviral treatment failure has emerged as a challenge in the management of pediatric human immunodeficiency virus (HIV) patients, especially in resource-limited countries despite accessibility to Highly Active Antiretroviral Therapy (HAART). A systematic review and meta-analysis was conducted to synthesize virological failure (VF) prevalence and ascertain its predictors in children in sub-Saharan Africa. An electronic database search strategy was conducted from January to September 2021 on PubMed, EMBASE, SCOPUS, HINARI, and CINAHL. Further, manual searching was conducted on non-indexed journals. Utilizing the JASP© version 0.17.2 (2023) statistical software, a meta-analysis of pooled prevalence of VF was estimated using the standardized mean differences. Further, selection models were used to assess the risk of bias and heterogeneity. The pooled odds ratios were estimated for the respective studies reporting on predictors of VF. The overall pooled estimate of the prevalence of VF in sub-Saharan Africa among the sampled population was 29% (95% CI: 27.0-32.0; p<0.001). Predictors of VF were drug resistance (OR: 1.68; 95% CI: 0.88-2.49; p < 0.001), poor adherence (OR: 5.35; 95% CI: 5.26-5.45; p < 0.001), nevirapine (NVP)-based regimen (OR: 5.11; 95% CI: 4.66-5.56; p < 0.001), non-usage of cotrimoxazole prophylaxis (OR: 4.30; 95% CI: 4.13-4.47; p < 0.001), higher viral load at the initiation of antiretroviral therapy (ART) (OR: 244.32; 95% CI: 244.2-244.47; p <0.001), exposure to the prevention of mother to child transmission (PMTCT) (OR: 8.02; 95%CI: 7.58-8.46; p < 0.001), increased age/older age (OR: 3.37; 95% CI: 2.70-4.04; p < 0.001), advanced World Health Organization (WHO) stage (OR: 6.57; 95% CI: 6.17-6.98; p < 0.001), not having both parents as primary caregivers (OR: 3.01; 95% CI: 2.50-3.53; p < 0.001), and tuberclosis (TB) treatment (OR: 4.22; 95% CI: 3.68-4.76; p <0.001). The mean VF prevalence documented is at variance with studies in other developing countries outside the sub-Saharan region. The high prevalence of HIV cases contrasting with the limited expertise in the management of pediatric ART patients could explain this variance.

## Introduction

Human immunodeficiency virus (HIV) remains a major global public health problem with people living with HIV/AIDS being estimated at 38.0 million as of December 2019 [1]. However, the introduction of Highly Active Antiretroviral Therapy (HAART) has reduced HIV/AIDS-related morbidity and mortality, hence improving the quality of life of people living with HIV/AIDS [2]. The newly proposed 95-95-95 strategy targets achieving viral suppression in 95.0% of HIV-positive patients on sustained HAART [1]. In 2019, an estimated 81% of all people living with HIV knew their status with 67% receiving HAART of which 59% achieved viral suppression [1]. In addition, 53% of children living with HIV globally received lifelong antiretroviral therapy (ART) [1]. The number of children living with HIV accessing HAART has increased drastically [3]. This has improved survival rates in pediatric HIV-positive patients including in sub-Saharan Africa [3].

However, ART treatment failure has emerged as a challenge in pediatric HIV patients on HAART [4]. Pediatric ART treatment failure rates of 19.3% to over 32% in resource-limited countries including sub-Saharan Africa have been reported [5]. Virological failure (VF) is a plasma viral load above 1000 copies/ml in an HIV patient after two consecutive viral load measurements 3 months apart, despite adherence to HAART for at least 6 months [6]. Viral load (VL) monitoring for all patients on HAART is the most accurate available measure of the effectiveness of treatment response and a key in diagnosing treatment failure [1]. Some associated factors with VF include poor adherence [7], inadequate dosing, and viral resistance [8]. However, little is known about the magnitude of HIV virological treatment failure and its predictors in pediatric patients in sub-Saharan Africa. Therefore, this review discusses the prevalence of VF and associated factors in children aged above 6 months and adolescents on HAART in sub-Saharan Africa. Furthermore, the study provides proportionate effects of the associated factors of VF.

## Methods

**Registration of review:** this review was registered in Prospero with registration: PROSPERO 2021 CRD42021230120.

**Eligibility criteria:** all cross-sectional, case-control, cohort, and randomized controlled studies conducted in sub-Saharan Africa documenting the prevalence and associated factors influencing VF in children above 6 months and adolescents on the treatment of HIV/AIDS were selected. The studies were reported in English and had a definable characterization of VF. Further, Studies outside the sub-Saharan African region or focused on non-HIV/AIDS-related treatment failure, studies that sampled adults, reviews, health demographic surveys, and editorials were not included.

**Information sources:** an electronic database search strategy was conducted from January 2021 to September 2021 on PubMed, EMBASE, SCOPUS, HINARI, and CINAHL. Further, manual searching was conducted on non-indexed journals: Web of Science, IBSS, BioMed Central, Directory of Open Access Journals (DOAJ), WHO electronic Library of Evidence for Nutrition Actions, and Google Scholar. The search for grey literature was conducted by contacting experts in retroviruses and opportunistic infections, following the reference list for potential articles and abstracts from HIV/AIDS scientific conferences.

**Search strategy:** the search terms: (“HIV” or “AIDS” or “human immunodeficiency syndrome” or “human immunodeficiency virus”) and (“predictor*” or “risk factor*” or “aetiology*” or “cause*”) and (“antiretroviral therapy” or “ART” or “highly active antiretroviral therapy” or “HAART”) and (“treatment failure” or “resistance” or “drug failure” or “viral treatment failure” or “virological failure” or “drug toxicity” or “poor adherence”) and (“child*” or “infant*” or “neonate*” or “adolescent*”) and (“sub-Saharan Africa*” or “Africa*” or “Zambia” or “Malawi” or “Mozambique” or “South Africa” or “Lesotho” or “Eswatini” or “Seychelles” or “Madagascar” or “Uganda” or “Tanzania” or “Senegal” or “Ethiopia” or “Nigeria” or “Mali” or “Zimbabwe” or “Ghana” or “Togo” or “Burkina Faso” or “Sierra Leone” or “Sudan” or “South Sudan” or “Botswana” or “Democratic Republic of Congo” or “Guinea” or “Niger” or “Guinea-Bissau” or “Burundi” or “Angola” or “Central African Republic” or “Benin” or “Mauritania” or “Equatorial Guinea” or “Chad” or “Cabo Verde” or “Comoros” or “Liberia” or “Eritrea” or “Djibouti” or “Rwanda” or “Congo Brazzaville” or “Kenya” or “Somalia” or “Namibia” or “Cameroon” or “Côte d'lvoire” or “Gabon” or “Gambia” or “Sao Tome and Principe”).

**Selection process:** the articles retained after building up queries in the electronic databases were populated in RefWorks (2020) to remove duplicates. Two reviewers (NM and LL) independently retrieved the articles and screened them based on the title and abstract. The articles in doubt were further screened by a third review (PH) and any disagreements were resolved through discussions among the three reviewers. The primary outcome was plasma viral load above 1000 copies/ml based on two consecutive viral load measurements done 3 months apart in a patient who has been on HAART for at least 6 months. The secondary outcomes were the associative factors influencing VF.

**Data extraction and management:** data on study characteristics and statistical parameters deciphering correlation or causality were abstracted into Microsoft Excel Spreadsheet. Thus, a preconceived and standardized abstraction form was formulated based on the PRISMA [9] guidelines for conducting systematic reviews. Two independent reviewers (MN and LL) each populated the abstraction form with the name of authors, year of publication, the country where the study was conducted, the title of the research, study design, aim, sampling strategy, sample size, and prevalence, study setting, characterization of VF (i.e. measurement), and potential associative factors.

**Methodological quality assessment:** the Hoy [10] tool for assessing the risk of bias in prevalence studies was utilized to evaluate the methodological rigor of the potential studies. The two reviewers (NM and LL) independently evaluated the quality of the studies, and any discordancy was resolved through the third reviewer (PH). As assessment of the validity and reliability of the measurement tools, an indication of potential confounders, and the relevance of statistical analysis used were strictly followed in each of the studies which passed the inclusion criteria. Further, the studies were ranked based on meeting the quality checks in the Hoy [10] tool in order to ascertain the risk of bias.

**Data synthesis and analysis:** to statistically assess the pooled effects of VF among pediatric patients on HAART, a meta-analysis was conducted to provide an overall analysis for prevalence and predictors of VF, subgroup analysis according to study design and region, and heterogeneity and risk of bias. The JASP © version 0.17.2 (2023) statistical software was utilized for the meta-analysis.

## Results

**Study selection:** the systematic electronic database searching strategy employed retained the following number of articles in each database: PubMed (n=502), EMBASE (n=123), SCOPUS (n=2), HINARI (n=13) and CINAHL (n=14). A manual searching strategy conducted on non-indexed journals retained hundred and twenty (n=120) articles; of which forty (n=40) were editorials, and fifty-three (n=53) were systematic reviews, thus, were immediately excluded. This brought the total number of articles retained after manual searching to twenty-seven (n=27). Combining the articles obtained from manual searching with those from systematic electronic database searching brought the total number of articles retained to six hundred and eighty-one (n=681). However, thirty-eight (n=38) articles were duplicates and were, therefore removed. Two investigators (MN and LL) further conducted independently the title and abstract screening of six hundred and forty-three (n=643) articles.

**Figure 1:**
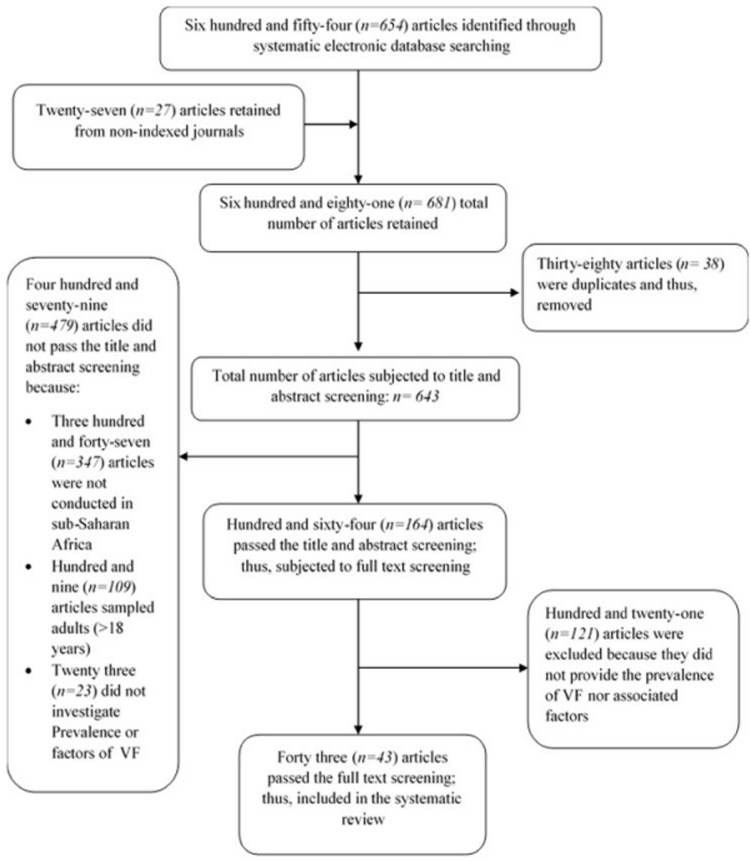
flow chart illustrating the study selection process for the systematic review and meta-analysis of the prevalence and predictors of virological failure among pediatric patients on HAART in sub-Saharan Africa

One hundred sixty-four (n=164) articles passed the title and abstract screening while four hundred and seventy-nine (479) failed. Three hundred and forty-seven (n= 347) articles were conducted outside sub-Saharan Africa, one hundred and nine (n= 109) articles sampled study participants outside the pediatric age group and/or had a mixture of children and adults as study participants while twenty-three (n= 23) did not clearly state the prevalence or predictors of VF in children. The articles which passed the title and abstract screening (n=164) were further subjected to full-text screening against the inclusion criteria by two investigators (MN and LL). Forty-four (n=44) articles were within the inclusion criteria and included in the systematic review. The one hundred and twenty-one (n=121) articles that were excluded after a full-text screening did not highlight any prevalence of VF and/or its predictors in children. The reference list of the articles which passed the full-text screening was also checked for relevant articles. However, no article was obtained through this process. The electronic and manual search process is illustrated in the flow diagram in Figure 1.

**Study characteristics:** concerning geographical location, sixteen were conducted in southern Africa [11-26], twenty-one in East Africa [4,27-46]; while West Africa and Central African regions accounted for six [47-52] and one [53] respectively. Of the sixteen studies in Southern Africa, nine were conducted in South Africa [11-18,25], one in Zimbabwe [19], two in Malawi [20,26], one in Zambia [21], two in Botswana [22,23] and one in Swaziland [24]. For East Africa, of the twenty-one, three were done in Tanzania [27-29], one in Rwanda [30], six in Kenya [31-36], eight in Ethiopia [4,37-40,43,45,46] and three in Uganda [41,42,44]. For west Africa, one from Ghana [47], three from Nigeria [48,49,52], and two from Cameroon [50,51]. One research study was conducted in the Central African Republic [53] (Table 1, Table 1 (suite)). Various methodological approaches were used in the studies included in the review, twenty-nine were cohort studies [11-13,15,17,18,20,21, 23,28-31,34-44,47-49,51,52], ten cross-sectional studies [4,16,19,22,24,27,45,46,50,53], two case control [23,32], and three clinical trials [14,25,33] (Table 1, Table 1 (suite)).

**Table 1:** characteristics of the 44 included studies reporting either the prevalence or the predictor(s) of virological failure among pediatric patients on HAART in sub-Saharan Africa

Region	Author (s) and year	Country	Study design	Sample size	Prevalence (%)	Valid/appropriate analysis
Western Africa	Barry *et al*. 2013	Ghana	Cohort	90.0	16.7	Yes
	Owuse *et al*. 2017	Ghana	Cohort	188.0	15.7	Yes
	Boerma *et al*. 2016	Nigeria	Cohort	82.0	32.9	Yes
	Ebonyi *et al*. 2014	Nigeria	Cohort	580.0	22.5	Yes
	Zoufaly *et al*. 2013	Cameroon	Cross-sectional	580.0	18.8	Yes
	Nlend *et al*. 2017	Cameroon	Cohort	375.0	17	Yes
Southern Africa	Barth *et al*. 2011	South Africa	Cohort	101.0	7	Yes
	Davies *et al*. 2011	South Africa	Cohort	5485.0	19.3	Yes
	Meyers *et al*. 2011	South Africa	Cohort	2795.0	16.3	Yes
	Rossouw *et al*. 2015	South Africa	Cohort	65.0	49	Yes
	Taylor *et al*. 2011	South Africa	Clinical trial	323.0	17	Yes
	Zanoni *et al*. 2011	South Africa	Cohort	555.0	13	Yes
	Brittain *et al*. 2018	South Africa	Cross-sectional	474.0	30	Yes
	Teasdale *et al*. 2018	South Africa	Cohort	397.0	16	Yes
	Jobanputra *et al*. 2015	Swaziland	Cross-sectional	835.0	46	Yes
	Lowenthal, *et al*. 2013	Botswana	Cross-sectional	804.0	32.2	Yes
	Huibers *et al*. 2018	Malawi	Cohort	35.0	66	Yes
	Tweya *et al*. 2019	Malawi	Cohort	1312.0	16	Yes
	van Dijk *et al*. 2011	Zambia	Cohort	267.0	11.5	Yes
	Makadzange *et al*. 2015	Zimbabwe	Cross-sectional	599.0	30.6	Yes
East Africa	Bitwale *et al*. 2021	Tanzania	Cross-sectional	300.0	34	Yes
	Dow et al., 2014	Tanzania	Cross-sectional	161.0	24	Yes
	Muri *et al*. 2017	Tanzania	Cohort	213.0	24.5	Yes
	Haile and Berha, 2019	Ethiopia	Cohort	318.0	8.3	Yes
	Yihun *et al*. 2019	Ethiopia	Cohort	402.0	49	Yes
	Sibhat *et al*. 2020	Ethiopia	Cohort	308.0	18.2	Yes
	Bayleyegn *et al*. 2021	Ethiopia	Cross-sectional	253.0	19.4	Yes
	Gelaw *et al*. 2021	Ethiopia	Cross-sectional	399.0	14.8	Yes

**Table 1(suite):** characteristics of the 44 included studies reporting either the prevalence or the predictor(s) of virological failure among pediatric patients on HAART in sub-Saharan Africa

Region	Author(s) and year	Country	Study design	Sample size	Prevalence (%)	Valid/appropriate analysis
East Africa	Tadesse *et al*. 2019	Ethiopia	Cohort	94.0	38.3	Yes
	Tadesse *et al*. 2021	Ethiopia	Cohort	484.0	15	Yes
	Osman and Yizengaw, 2020	Ethiopia	Cohort	140.0	11	Yes
	Humphrey, *et al*. 2019	Kenya	Cohort	7667.0	38	Yes
	Kadima *et al*. 2019	Kenya	Case-control	1190.0	37	Yes
	Lehman, *et al*. 2012	Kenya	Clinical trial	20.0	35	Yes
	Sivapalasingam et al., 2014	Kenya	Cohort	626	-	Yes
	Wamalwa *et al*. 1999	Kenya	Cohort	100.0	34	Yes
	Kabogo *et al*. 2017	Kenya	Cohort	146.0	24	Yes
	Huibers *et al*. 2018	Uganda	Cohort	286.0	38.8	Yes
	Kityo *et al*. 2017	Uganda	Cohort	287.0	32.1	Yes
	Sebunya *et al*. 2013	Uganda	Cohort	701.0	34	Yes
	Mutwa *et al*. 2014	Rwanda	Cohort	123.0	16	Yes
	Kuhn *et al*. 2018	South Africa	Clinical trial	154	-	Yes
Central Africa	Mossoro-Kpinde *et al*. 2017	Central African Republic	Cross-sectional	220.0	60	Yes
Multi-centre study	Boender et al., 2016	Multi-centre study	Cohort	289.0	29.5	Yes

### Meta-analysis

**Figure 2:**
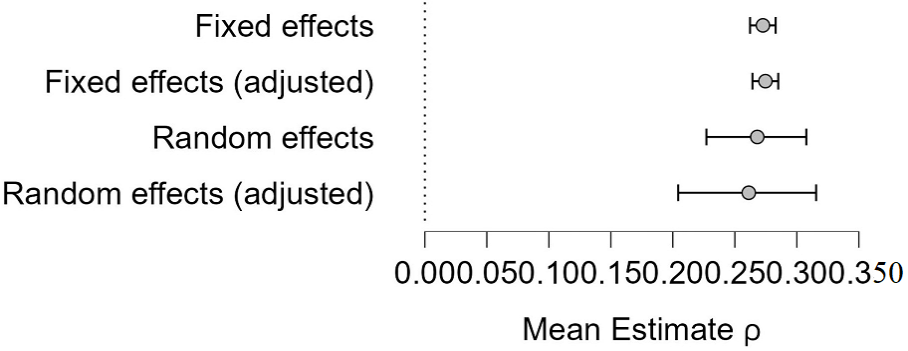
mean model estimates assessing heterogeneity through the standardized estimated effects from included studies (42 studies on prevalence)

**Figure 3:**
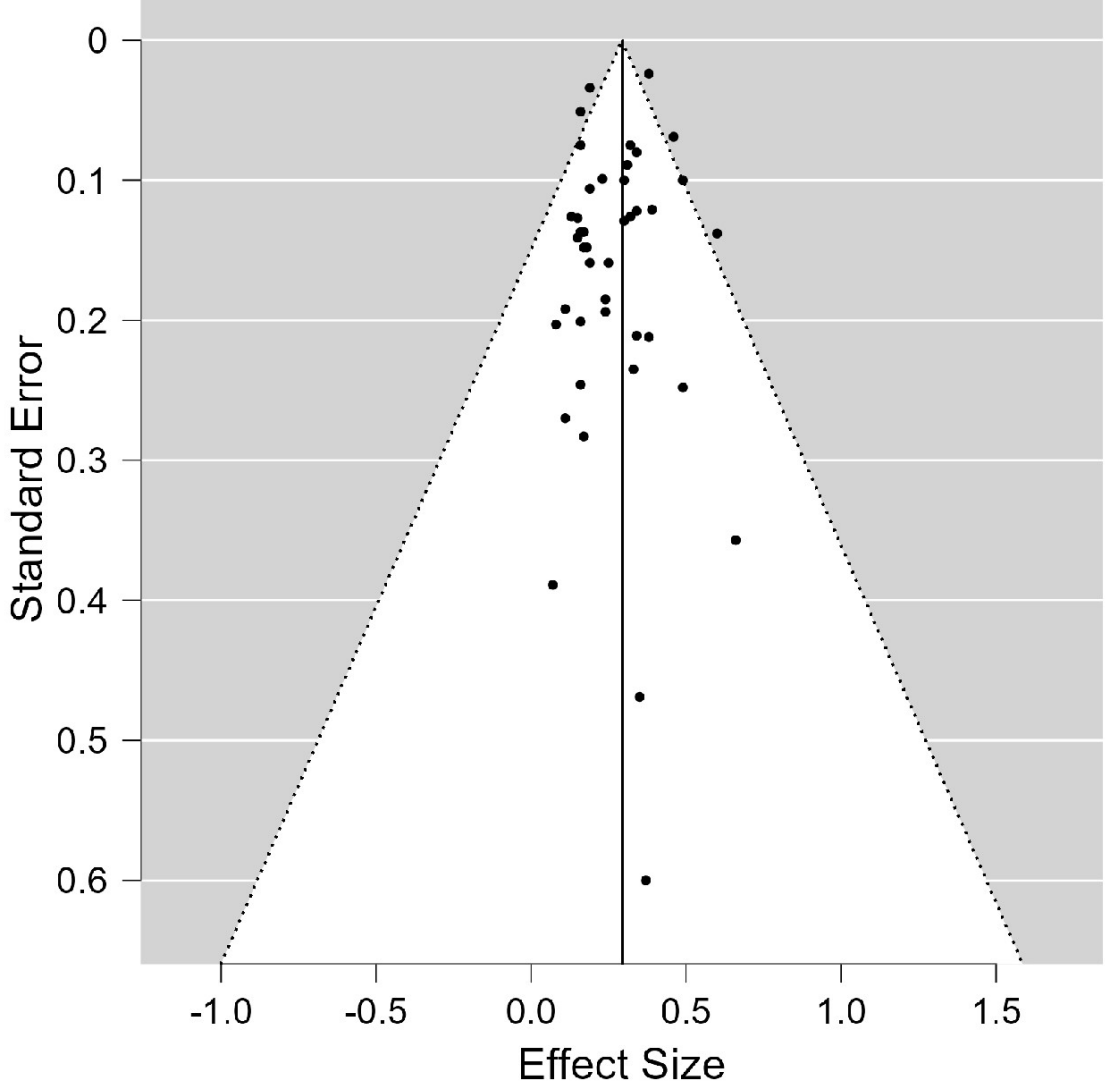
funnel plot for publication bias, Egger's regression test (p = 0.119) supporting the absence of publication bias using effect sizes on the x-axis and standard error on the y-axis

**Assessment of heterogeneity and publication bias:** utilizing selection models in JASP© 0.17.2 (2023) statistical software, the test for heterogeneity gives a Q value of 457.7 (p <0.001) signifying evidence for heterogeneity. Further, using the recommended p value threshold of 0.1 for the selection model, the analysis indicates no evidence of publication bias (p = 0.697). The mean model estimate is illustrated in Figure 2. In addition, the Egger´s regression test also supports the absence of publication bias (p = 0.119) as illustrated in the funnel plot in Figure 3.

**Figure 4:**
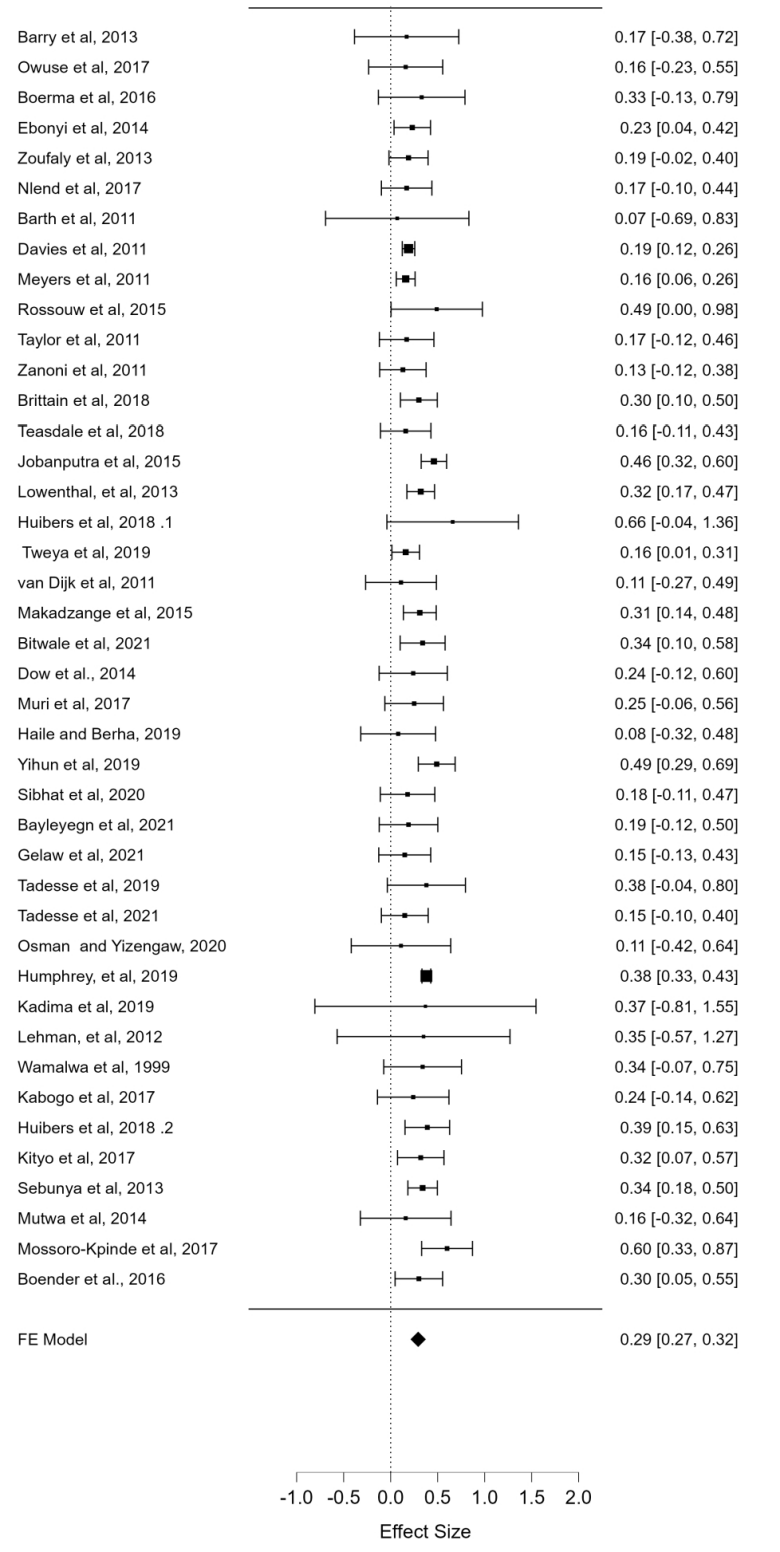
Forest plot of the prevalence (effect size) of virological failure with respective 95% confidence intervals from the 42 studies which estimated the prevalence (presented as ratios)

**Pooled prevalence of VF and subgroup analysis:** the overall mean prevalence of VF in sub-Saharan Africa among the sampled population was 29% (95% CI: 27.0-32.0; p<0.001). This was estimated from 42 studies, with a total sample population (N) of 30,043. This is presented in the forest plot in Figure 4. Considering the region, the subgroup analysis for prevalence of VF was: West Africa 21% (95% CI: 9.0-32.0; p < 0.001), East Africa 35% (95% CI: 31.0-39.0), Southern Africa 23% (95% CI: 19.0-27.0; p < 0.001), Central Africa 60% (95% CI: 53.0-66.0; p < 0.0001), and multicenter study 29.5% (95% CI: 23.7-35.9; p < 0.001). The subgroup analysis for study design was cohort 28% (95% CI: 25.0-31.0; p < 0.001), cross-sectional 34% (27.0-40.0; p < 0.001), clinical trial 19% (95% CI: -9.0-24.0; p = 0.187) and case-control 37% (95% CI: 34.0-40.0; p < 0.001).

**Influential analysis:** an assessment of sources of heterogeneity indicated the absence of influential impact among the 42 studies considered for pooled estimates of prevalence. Thus, there were no significant changes in the fit models that were indicative of influencing heterogeneity.

**Predictors of virological failure (VF):** the predictors of VF were documented in 37 studies included in this review. These were drug resistance, poor adherence, NVP-based regimen, non-usage of cotrimoxazole prophylaxis, higher viral load at the initiation of ART, exposure to PMTCT, increased age/older age as a risk factor for VF, advanced WHO stage, not having both parents as primary caregivers, and TB treatment.

**Figure 5:**
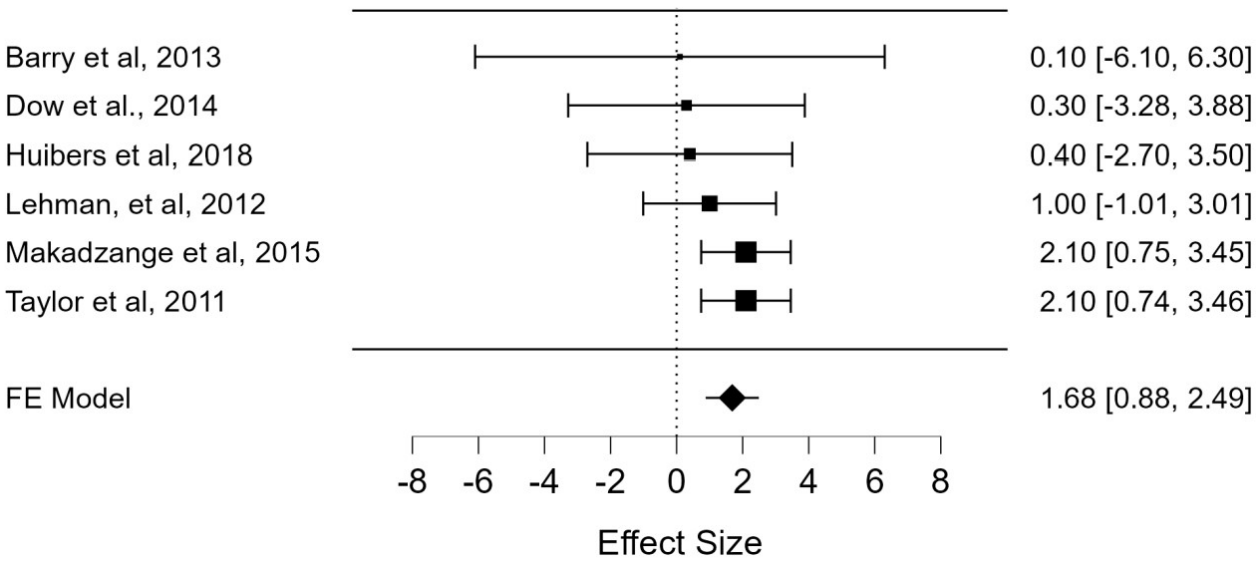
the pooled effects of drug resistance mutation (DRM) on virological failure from 6 studies with a total sample population (N) of 1,479 pediatric patients in sub-Saharan Africa

**Drug resistance mutation (DRM):** drug resistance was documented as a major predictor of VF whether acquired or pre-drug resistance affecting mainly non-nucleoside reverse transcriptase inhibitors (NNRTIs), nucleoside reverse transcriptase inhibitors (NRTIs), and protein inhibitors (PIs). This was reported in six studies [14,19,28,33,44,47] with a total sample population (N) of 1479. A pooled odds ratio of 1.68 (95% CI: 0.88-2.49; p < 0.001) was indicative that DRM likely influenced VF in the sampled population (Figure 5).

**Figure 6:**
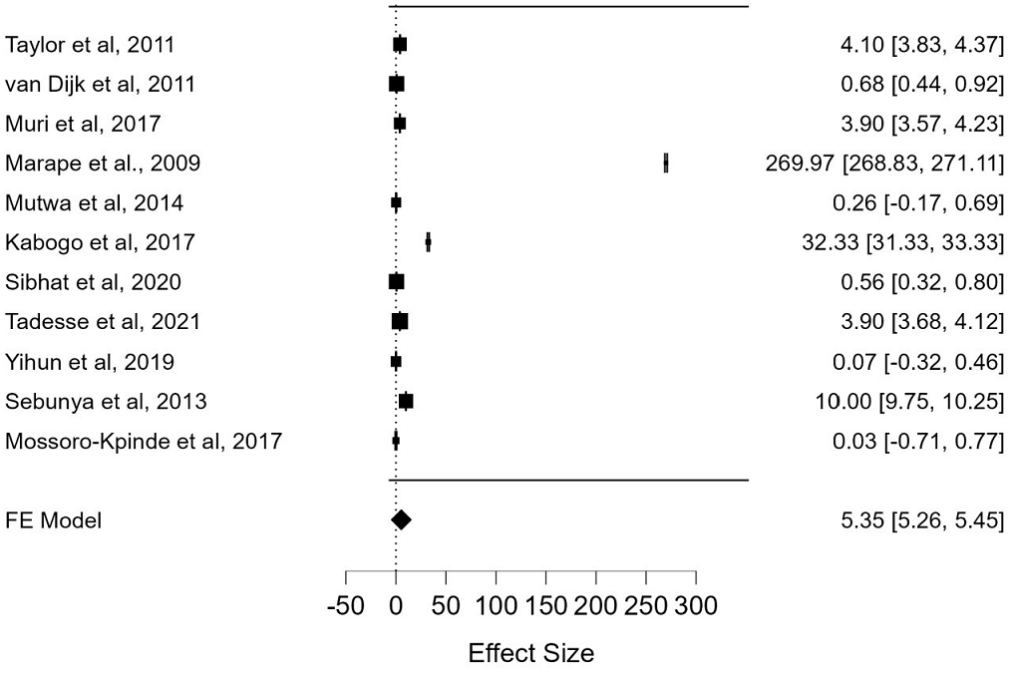
the pooled effects of poor adherence on virological failure from 11 studies with a total sample population (N) of 3,928 pediatric patients in sub-Saharan Africa

**Poor adherence:** of the 44 studies considered in this review, eleven studies [14,21,23,29,30,36,38-40,44,53] documented poor adherence to HAART as a predictor of VF from a sample population of 3928. Study participants with poor adherence were more likely to have VF when compared to those who had good adherence. This was statistically indicated by a pooled odds ratio of 5.35 (95% CI: 5.26-5.45; p < 0.001) (Figure 6).

**Figure 7:**
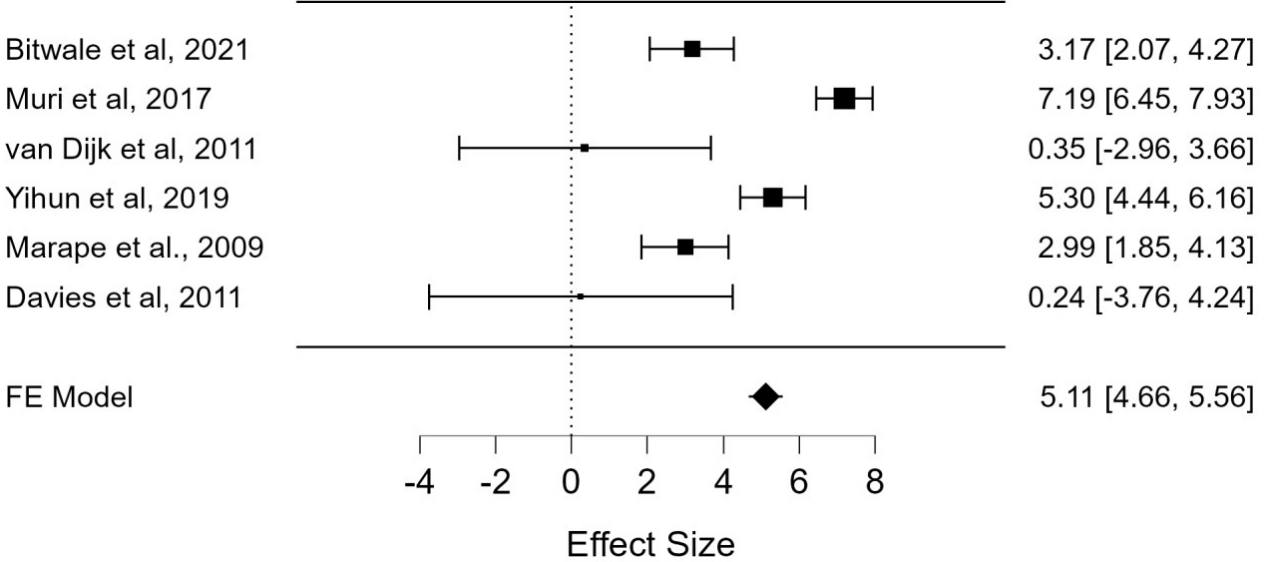
the pooled effects of nevirapine-based regimen (NVP) on virological failure from 6 studies with a total sample population (N) of 7,408 pediatric patients in sub-Saharan Africa

**NVP-based regimen:** nevirapine-based regimen (NVP) has been documented as predictor of VF in six studies [12,21,23,27,29,40] from a sample population of 7408. A pooled odds ratio of 5.11 (95% CI: 4.66-5.56; p < 0.001) was indicative of the significant influence that NVP-based regime has on VF (Figure 7).

**Non-usage of cotrimoxazole prophylaxis:** it has been established that non-usage of cotrimoxazole prophylaxis likely increases the occurrence of VF. In two studies [27,49] with a sample population of 880, a pooled odds ratio of 4.30 (95% CI: 4.13-4.47; p < 0.001) was indicative of the significant influence of the non-usage of cotrimoxazole prophylaxis has on VF.

**Higher viral load at the initiation of ART:** a viral load of >1000 copies/ml at ART initiation was an important predictor of VF. It has been demonstrated that children with an initial viral load > 1000 copies are more likely to experience VF. In five studies [12,23,24,30,48] with a sample population of 7266, a pooled odds ratio of 244.32 (95% CI: 244.2-244.47; p <0.001) indicated the significant influence of higher viral load at ART initiation on VF.

**Exposure to PMTCT:** the review also documented exposure to prevention of mother to child transmission especially when NVP regime is used likely influenced VF. This was documented in four studies [12,23,30,41] with a sample population of 7050 and a pooled odds ratio of 8.02 (95%CI: 7.58-8.46; p < 0.001).

**Increased age/older age as a risk factor for VF:** adolescent age was associated with an increased risk of VF. This was reported in three studies [16,22,28] with a sample population of 1439 and a pooled odds ratio of 3.37 (95% CI: 2.70-4.04; p < 0.001).

**Advanced WHO stage:** the advanced WHO HIV clinical stage was associated with an increased risk of VF. This was reported in six studies [4,24,37,38,42,54] with a sample population of 2290 and a pooled odds ratio of 6.57 (95% CI: 6.17-6.98; p < 0.001).

**Not having both parents as primary caregivers:** interestingly, not having both parents as primary caregivers was a major predictor of VF. It was documented that HIV-positive children whose both parents died were likely to experience VF. A motherless orphan had a higher risk of experiencing VF compared with those whose mothers were alive. This was reported in five studies [31,34,37,51,54] with a sample population of 9388 and a pooled odds ratio of 3.01 (95% CI: 2.50-3.53; p < 0.001).

**A child being on TB treatment:** concomitant treatment of tuberculosis in an HIV-infected child on HAART can lead to VF. In addition, children who had tuberculosis at baseline were more likely to have VF. This was reported in four studies [14,15,31,38] with a sample population of 8388 and pooled odds ratio of 4.22 (95% CI: 3.68-4.76; p <0.001).

## Discussion

The findings of this review indicate a mean prevalence of 29% of VF among children aged below 15 years in sub-Saharan Africa. The regional analysis ranked West Africa (21%) with the lowest VF pooled estimate rate while Central Africa (60%) had the highest. However, it is important to highlight that only one study was considered from Central Africa. Therefore, the reported mean value might not reflect the picture across the Central African region. The predictors of VF identified in this systematic review and meta-analysis were drug resistance, poor adherence, NVP-based regimen, non-usage of cotrimoxazole prophylaxis, higher viral load at the initiation of ART, exposure to PMTCT, increased age/older age as a risk factor for VF, advanced WHO stage, not having both parents as primary caregivers, and TB treatment.

The findings of this review are at variance with what has been documented in studies in other developing countries outside the sub-Saharan region. In Thailand, Cambodia, and India, the mean VF ranged from 3.3 to 27% in children treated for HIV 1 using the WHO standard first-line ART for a period not less than six months [55-59]. This highlights a higher success rate in the management of HIV compared to rates obtained in sub-Saharan Africa. Differences in settings could explain this variance since sub-Saharan Africa accounts for most HIV cases coupled with several challenges in the management of pediatric ART patients such as limitations in resources availability and lack of expertise in the management of pediatric ART patients.

Concerning the predictors of VF, immunosuppression and high VL at the initiation of HAART were documented. In agreement with this review, several studies have shown the association of better HAART response with less HIV-1-associated immunodeficiency and lower HIV RNA viral load at diagnosis and before starting HAART [55,56,58-60]. All these findings reinforce the importance of initiating an early HAART in HIV pediatric patient.

Furthermore, this review showed that poor adherence and increased age for patients on HAART were risk factors for VF. These findings are similar to other studies conducted in the United Kingdom and Asia [57,61]. Poor adherence has been long known to be associated with VF as missing doses of HAART are likely associated with reduced levels of drugs below the therapeutic levels in blood consequently leading to increased risk of VF. In addition, poor adherence in the adolescent age group remains common especially with older age likely associated with rebellious behavior leading to poor adherence-subsequently to resistance and VF. Comorbidity of TB in an HIV-positive patient on HAART was equally a predictor of VF. This finding is congruent with what was documented in pediatric populations in six countries in Asia [57], a setting that is similar to sub-Saharan Africa in terms of disease burden.

Caregivers have a critical role to play in the management of pediatric ART patients and this has a bearing on the ART outcomes. In this review, the non-availability of biological parents as primary caregivers has been associated with an increased risk of VF [34,37,40]. This finding is in consonant with a multicenter retrospective cohort study in the Asian-Pacific [59]. Having family members other than biological parents/grandparents as primary caregivers increased the risk of subsequent VF among Asian children living with HIV. Thus, it emphasizes that dedicated caregivers are critical in assuring care and adherence in children who are not able to fend for themselves. Another factor predictive of VF based on this review was advanced WHO stage [4,24,37,38,40,42], a finding that is consistent and associated with worsening immunodeficiency which in turn leads to advanced WHO stage. The advanced WHO stage is the tail end of the consequence of VF indirectly i.e. clinical failure.

Various studies in this review documented perinatal exposure to ARVs as a predictor of VF for patients who were put on the first-line ART drugs [12,23,30,41]. This was a common case for patients who received single doses of NVP without a tail-end cover which in turn raised the risk of resistance to the NNRTI-based regimen. These findings are in congruence with studies conducted in the United Kingdom (UK) which highlighted maternal exposure to ART perinatal increased the risk of VF were NVP based regimen [61].

In addition, this review has demonstrated drug resistance as a major contributor to VF with several studies documenting drug resistance mutations for both NNRTI and PI as significant risks for VF [28,40,42,44,47,53]. These findings are similar to a cohort study [62] in France in which the prevalence of resistance to any drug was 82.4%. Further, resistance ranged from 76.5% for NRTI, to 48.7% for NNRTI and 42.9% for PIs. Resistance to at least one drug of two classes and three classes (triple resistance) was 31.9% and 26.9%, respectively. These findings highlight drug resistance is one of the key factors contributing to VF in children and should be a priority concern in patients failing on ART.

## Conclusion

The mean VF prevalence documented is at variance with studies in other developing countries outside the sub-Saharan region. The high prevalence of HIV cases contrasting with the limited expertise in the management of pediatric ART patients could explain this variance. Health authorities should consider including in the HIV treatment guidelines routine testing for drug sensitivity pattern in HIV positive children as pediatric drug resistance has been documented on initiation of ART in various settings. Further, the component of screening and/or treating for opportunistic infections (OIs) must be strengthened to ensure the former are thoroughly and aggressively treated to reduce the risk of VF since OIs are a risk factor for treatment failure. While it is true that adherence is part of the routine in care for HIV patients, strong re-emphasis should be placed on adherence as well since poor adherence has been linked to high rates of VF.

### What is known about this topic

- *A higher prevalence of VF among adults living with HIV has been documented in sub-Saharan Africa;*
- *Poor adherence, TB co-infection, late ART initiation, and CD4+ count have been highlighted as predictors of VF in adults living with HIV*.

### What this study adds

- *Provides the regional mean prevalence of VF among pediatric patients on HAART in sub-Saharan Africa;*
- *Documents a comprehensive summary of predictive factors of VF among pediatric patients in sub-Saharan Africa*.
